# Community-based sustainable vector management strategies in rural areas of Zhejiang Province: criteria development and long-term impact assessment of vector control toward “Four Pest-Free Village” pilot program

**DOI:** 10.3389/fvets.2025.1445755

**Published:** 2025-06-06

**Authors:** Jinna Wang, Jun Xia, Wenrong Zhang, Tianqi Li, Mingyu Luo, Qinmei Liu, Junxiang Guo, Jimin Sun, Zhenyu Gong, Jianmin Jiang

**Affiliations:** ^1^Zhejiang Provincial Center for Disease Control and Prevention, Hangzhou, China; ^2^Zhejiang Patriotic Health Development Center, Hangzhou, China; ^3^Hangzhou Medical College, Hangzhou, China

**Keywords:** vector, mosquito, fly, rodent, cockroach, density, village

## Abstract

**Objectives:**

This study aimed to develop the relevant criterion of pilot program of the “Four Pest-Free Village”(FPFV) and to further investigate the effects on vector control in Zhejiang Province.

**Methods:**

The criterion of the FPFV was developed based on actual pilot experience and expert consultations. Vector density surveillance was conducted in all 11 prefecture-level cities of Zhejiang Province, including two FPFV and two control villages for each city. The CDC light trap method and Breteau Index (BI) method were used to monitor the density of mosquito. The fly trap method, night trapping method, and sticky board method were used to monitor the density of fly, rodent, and cockroach, respectively. Surveillance for mosquitoes and flies was conducted monthly from April to November 2023. Rodent and cockroach monitoring was conducted every odd month. Kruskal–Wallis test was used for data analysis.

**Results:**

The FPFV evaluation covered village organization management, health education, vector integrated control method, vector surveillance, and vector density control levels. A village could be named FPFV if the whole scored over 80 points. The *Culex pipiens pallens* (91.03%) was major species of mosquitoes; the total BI was 14.25; the *Sarcophagidae* (31.97%) and *Musca domestica* (31.41%) were major species of flies; the *Rattus norvegicus* (38.56%) was major species of rodents; the *Blattella germanica* (65.68%) was major species of cockroaches in rural areas of Zhejiang Province. All the vector densities investigated in the FPFV were lower than controls, including the adult mosquitoes (2.32 vs. 4.87 mosquitoes per trap-night), mosquito larvae (BI: 9.70 vs. 19.41), flies (1.32 vs. 2.17 flies per trap), rodents (0.41 vs. 0.84 rodents per 100 trap-nights), and cockroaches (the cockroach capture rate: 1.77% vs. 5.87%), and all the results were statistically significant (*p* ≤ 0.05).

**Conclusion:**

The findings from this study indicated that the FPFV was suitable for rural areas, and the method of sustainable vector management strategies was practical and reliable. Vector density in the FPFV could be controlled at a low level for a relatively long time than control village, which could greatly reduce the harassment of vector species on the population and the spread of related diseases.

## Introduction

The mosquito, fly, rodent, and cockroach, commonly known as the “four pests” in China (1), can affect people’s health by harassing people and spreading related diseases (2–4). Research found that more than 80% of the global population were at risk of vector-borne disease, in which mosquito-borne disease was the largest contributor to human vector-borne disease burden (5). Mosquito could transmit chikungunya, dengue, Japanese encephalitis, malaria, West Nile, yellow fever, Zika, etc. (6). Rodents could transmit several bacterial and viral infectious diseases such as hemorrhagic fever with renal syndrome, leptospirosis, and plague (7). The fly and cockroach played an important role in the mechanical transmission of the food-borne pathogens, including *Salmonella* spp., *Escherichia coli* O157: H7, and *Shigella* spp., resulting in increased risk of diarrheal disease (8, 9). Zhejiang Province is located in the southeast of China, with the subtropical monsoon climate, which is suitable for the survival of vectors, especially the “four pests,” always has a long-term prevalence of vector-related infectious diseases and an extremely high vector-borne infectious disease burden. For example, during 2004 to 2018, a total of 1,654 indigenous dengue cases and 12 outbreaks were reported in Zhejiang Province (10). The direct and indirect economic burdens of dengue patients were estimated at USD 405,038 and USD 140,364, respectively, while the total annual cost for the government and organizational sectors to prevent and control dengue fever was estimated to be USD 7,075,654 in 2019 of Zhejiang Province (11). The positive detection of dengue virus in mosquito specimens collected during the dengue fever outbreak in Zhejiang Province highlights the crucial role of mosquito surveillance in the prevention and control of dengue fever (12). Similarly, among rodent samples collected in Zhejiang Province in 2022, the positive rates were 12.28% for *Leptospira*, 1.00% for hantavirus, and 0.15% for *Orientia tsutsugamushi* (13). This indicated that rodent-borne diseases in Zhejiang Province were in a relatively severe situation. Thus, prevention and control of the “four pests” epidemic was of great significance to reduce the occurrence of related infectious diseases.

In recent years, with the development of the Patriotic Health Campaign in China, the density of the “four pests” had been sharply decreased. However, it was relatively lagging behind in the rural areas, with a large number of vector breeding ground and a relatively high vector density (14). From this perspective, the vector management strategies in rural areas through economic and effective ways should always been a focus. From 2016, the Zhejiang Provincial Center for Disease Control and Prevention (CDC) began to explore a rural mosquito sustainable control model with local characteristics of southern parts of China, known as the “Mosquito-Free Village” (15). On this basis, the pilot of the “Fly-Free Village” was carried out to explore the sustainable control technologies for the fly in rural areas in 2019 (16). The successful pilot projects provided a good idea for the sustainable control technology of the “four pests” in rural areas. From 2020, Zhejiang CDC began to promote the pilot projects of the “Four Pest-Free Village” (FPFV) (17), with the support from Zhejiang Patriotic Health Campaign Committee office. The FPFV was a series of villages, where the density of the “four pests” was persistently controlled at an extremely low level, without disturbing human’s life and reducing the occurrence of infectious diseases related to these vectors. Focusing on improving the living environmental condition of rural residents and further consolidating the foundation of rural vector disease prevention and control had become one of the important strategies to promote the patriotic health movements in rural areas of China.

By the end of 2023, the pilot program of the FPFV in Zhejiang Province had been persistent for 4 years, and the FPFV had reached a huge number. It had formulated a relatively mature evaluation system after constant adjustments and experiments, which put a positive effect on the density control of the “four pests” (18). In this study, we summarized the pilot experience and developed the relevant standards for the evaluation of the FPFV. A sampling survey was also adopted in 2023 to monitor the vector density in the FPFV and the control villages, to evaluate the actual long-term vector control effects.

## Materials and methods

### The pilot method of the FPFV

The vector control work organization was established. The role of communities was fully leveraged in the sustainable control of the vectors, and a rural vector control work organization was established under the leadership of the village committee. The work organization should formulate plans including the responsibilities of participants, vectors surveillance and control methods, evaluation of the control effects, and health education for the villagers.

Health education. The vector control work organization or the volunteer team trained the villagers by means of health lectures, on-site demonstration, posting slogans, leaflets, online chat group, and other interactive methods, to enhance the villagers’ health literacy of scientific vector control.

Vector control. The work organization mobilized the villagers to participate in the activities of vector control, advocating for comprehensive control measures that focus on environmental control methods, combining physical and biological control methods, and chemical control methods if necessary. Environmental control methods mainly included thoroughly carrying out village sanitation cleanup, implementing road hardening, rectifying green belts, and clearing indoor and outdoor clutter without leaving any hygiene neglected area; turning over pots and cans, placing ground containers such as wine bottles and earthenware jars indoors or covering them, changing the water weekly, or removing any standing water from containers for aquatic plants and trays under potted landscapes; properly handling garbage, with easily decomposable waste being collected separately and processed daily; and always covering food for storage, or other effective environmental sanitation measures. Physical control mainly involved using devices such as mosquito light traps, fly traps, mousetraps, and cockroach sticky traps to eliminate the “four pests,” improving facilities by sealing gaps in walls, and installing floor drains, rat guards, and rodent-proof nets in key areas such as kitchens and storerooms, as well as fitting windows and doors with screens, or employing other barrier methods such as ultrasonic repellents and electric grids to prevent the entry of these pests. Biological control mainly includes raising fish such as *Gambusia affinis*, *Cyprinus carpio,* or *Cobitis taenia* in village water bodies or paddy fields for mosquito control, raising cats for rodent control, and employing biocontrol agents such as *Bacillus thuringiensis israelensis* (Bti) and *B. sphaericus* for mosquito control. Chemical control was generally not recommended in the FPFV. However, it was still necessary for breeding sites that were difficult to clean or when other control measures failed to achieve satisfactory results. Pyrethroid insecticides such as permethrin, beta-cypermethrin, and deltamethrin, as well as a few carbamate insecticides and organophosphorus insecticides, were recommended for the control of adult mosquitoes and flies. Organophosphorus insecticides such as temephos, in combination with insect growth regulators such as pyriproxyfen and S-methoprene, were recommended for the control of mosquito larvae. For cockroach control, gel baits containing insecticides such as fipronil, dinotefuran, and imidacloprid were recommended. For rodent control, anticoagulant rodenticides such as bromadiolone and brodifacoum were recommended as baits.

A long-term mechanism was established for density monitoring and control. It was recommended to conduct routine breeding site cleaning work once a week. During the active seasons for mosquito and fly (April to November in Zhejiang Province), the mosquito and fly density monitoring should be carried out once a month. Cockroach and rodent monitoring should be conducted once every odd-numbered month throughout the year. Vector control work should be carried out in combination with density monitoring work. For months with abnormal increases in vector density, it was suggested to widely mobilize the villagers to carry out thorough control efforts. The scope of vector control covered the village and its surrounding area within 50–100 meters, which would be ultimately determined based on the specific environment around the village. The vector density indicators were mainly referred to the “mosquito-free village” (15), the “fly-free village” (16), and the National Standard of the People’s Republic of China (PRC) (19–22).

Effect evaluation. In accordance with the principles of voluntariness, self-construction, and self-management, the role of the community was fully leveraged, and the village committee decided on its own whether to carry out FPFV. Villages that intended to establish FPFV needed to conduct a self-assessment of the effectiveness after implementing a series of measures, including establishing organizations, mobilizing villagers, conducting health education, and carrying out vector surveillance and control. If the self-assessment met the requirements, they could apply for FPFV assessment. The patriotic health department organized more than three experienced vector control experts to conduct on-site assessments in the year after the application was submitted.

### Criterion of the FPFV

The evaluation of the FPFV should encompass five aspects, namely, village organization management, health education, integrated vector control methods, vector surveillance, and vector density control levels. The experienced experts were invited to conduct and score the on-site assessments according to the criterion of the FPFV (Table 1). According to the criterion entitled “Guide of sustainable control and prevention of vectors in rural areas-the four pests” (18), and considering the actual pilot experience and expert consultations, a score of 80 points was explicitly established as the threshold for evaluating the effectiveness of FPFV. If the overall score was above 80 points, then it could be named the FPFV.

**Table 1:** Criterion of the FPFV.

Main contents	Specific components (assigned score)	Criterion for indicator evaluation (score)
Village Organization Management	Vector control work organization (6 points)	1. Establish a vector control work organization, with the principal responsible person of the village committee serving as the group leader. (2 points) 2. Adequate working funds (2 points). 3. The village regulations and agreements should include the contents of vector control (2 points).
	Daily management (6 points)	1. Establish a long-term management mechanism (3 points). 2. The construction plans should be formulated including vector surveillance and control plans etc. (3 points).
	Villagers satisfaction (5 points)	The satisfaction of the villagers for the FPFV≥90% (5 points).
Health education	Health education activities (15 points)	1. Set up prominent signs and health warning messages for vector control at the entrance or other prominent locations of the village (7 points). 2. Carry out health education activities at least four times each year, through health lectures, display boards, science popularization videos, short message, and village propaganda speakers, etc. (8 points).
	Health education content (5 points)	The content of the health education is scientific and easy to understand, including basic knowledge of the vectors and related infectious disease (5 points).
	Knowledge-Attitude-Behavior of the villagers (15 points)	1. The vector control knowledge awareness rate of the villagers≥90% (5 points). 2. The vector control behavior of the villagers≥85% (5 points). 3. The support rate of the villagers≥90% (5 points).
The vector integrated control methods	The environment improvement and the vector breeding site clean-up (10 points)	1. The overall environment of the village is clean and tidy (5 points). 2. There is no obvious uncleaned vector breeding ground in the village (5 points).
	The vector control methods (8 points)	1. The vector control methods include the environmental control method, the physical control method, the biological control method, and the chemical control method if necessary (4 points). 2. The control method is reasonable and does not cause safety risk to humans or poultry etc. (4 points).
The vector surveillance	The vector density surveillance (5 points)	Regular vector density surveillance is carried out, and the vector control measures are carried out timely when the surveillance results showed a high density (5 points).
The vector density control level	Control effect of the vector density and related infectious diseases (25 points)	1. No local vector-related infectious diseases or insecticide poisoning incidents. No complaints or reports from villagers (5 points). 2. The density of the four pests has reached a certain control level (20 points).

### The vector density control level of the FPFV

The vector density control levels of the FPFV are as follows. The mosquito density control index: BI <5.0; route index ≤0.5 (Route index method); landing index ≤0.5 (Landing index method). The fly density control index: fly density ≤0.5 flies/cage (Fly trap method); route index ≤0.5 (Route index method). The rodent density control index: the rodent density ≤1.0 per 100 trap-nights (Night trapping method); indoor rodents trace positive rate ≤3.0% (Rodents trace method); external environment route index ≤3.0 (Route index method). The cockroach density control index: the cockroach capture rate ≤1.0% (Sticky boards method). The infestation rate of adult cockroach was less than 1.0%, the detection rate of cockroach ootheca was ≤1.0%, and cockroach trace detection rate was ≤3.0% (visual inspection method).

### The long-term vector density control effect evaluation of the FPFV

**Figure 1:**
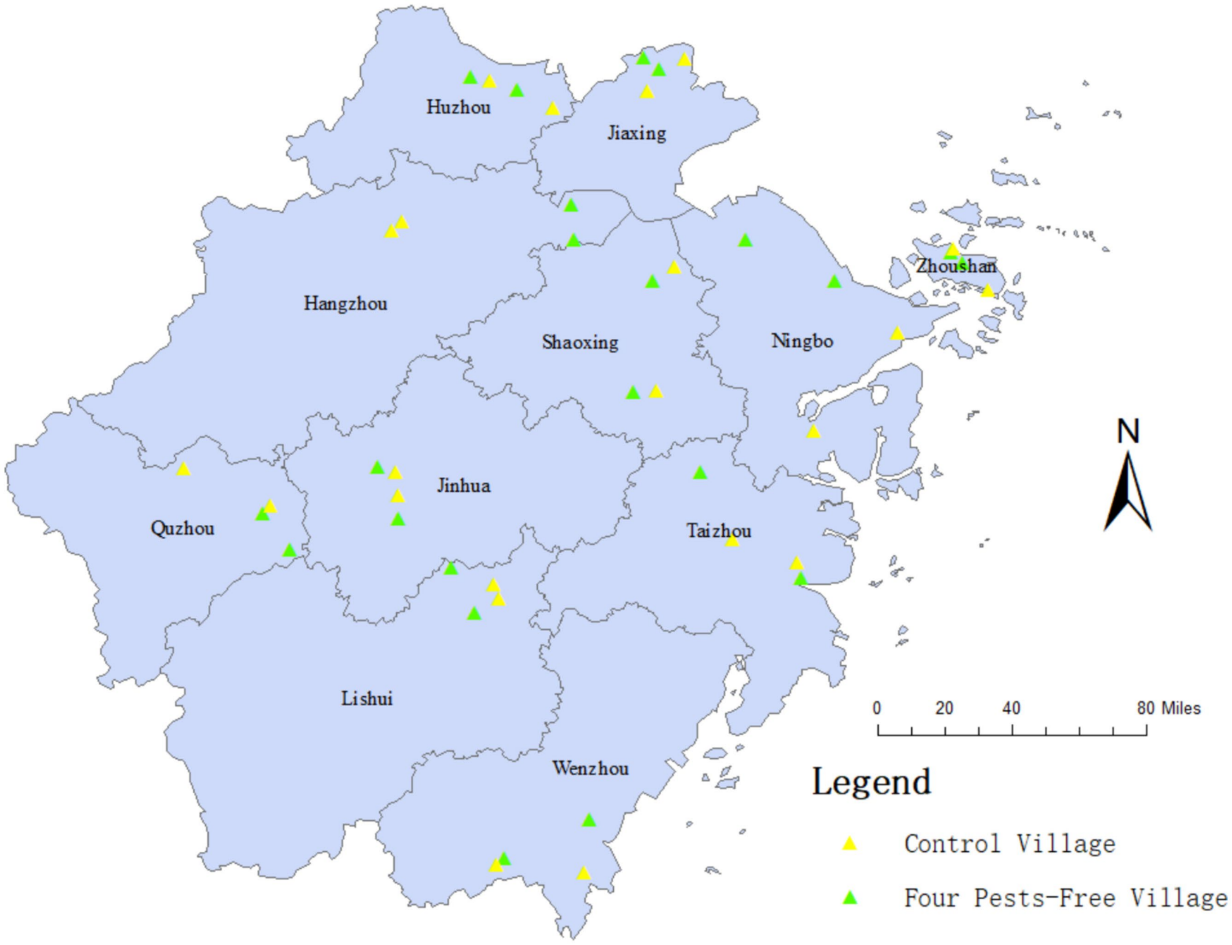
Distribution of sampled villages in Zhejiang Province.

To observe the long-term control effects on the pest density, the density surveillance was conducted in all the 11 prefecture-level cities of Zhejiang Province, namely, Ningbo, Wenzhou, Lishui, Taizhou, Quzhou, Zhoushan, Huzhou, Jiaxing, Shaoxing, Jinhua, and Hangzhou city, including all the habitat types encompassing mountains, hills, plains, basins, and islands to avoid selection bias from different regions. Two villages were selected from those that had been assessed and designated as FPFV by government agencies for at least 1 year in each city. In the same city or district, the principle of 1:1 matching was applied to select the control village (Figure 1). To avoid interference from other factors, the control village should be matched with the experimental village in terms of geographical area, landscape type, habitat type, village size, number of households, population size and demographics, infrastructure, socioeconomic status, ecosystem characteristics, and meteorological conditions. These detailed match measures would ensure that the control and experimental villages were comparable in key aspects, thereby minimizing potential confounding influences and providing a more robust basis for analysis. In addition, the control village should not have undergone any intervention of FPFV. To ensure that there are sufficient sample households for the monitoring of mosquitoes, flies, rodents, and cockroaches, all villages must meet the requirements of having more than 300 households and a permanent population greater than 1,000 people. According to the Law of the People’s Republic of China on Prevention and Treatment of Infectious Diseases, we have reviewed the National Notifiable Disease Surveillance System and confirmed that there were no vector-borne infectious diseases reported in the villages during the past 2 years.

Zhejiang Province has a typical subtropical monsoon climate, characterized by moderate temperatures, abundant precipitation, and distinct seasons, which are highly conducive to the survival and reproduction of vector organisms. Therefore, in selecting the monitoring time for this study, local climatic conditions and the seasonal fluctuations in vector population densities were taken into consideration. The monthly meteorological data were obtained from the National Tibetan Plateau Data Center. Given the relatively low temperatures in winter in Zhejiang Province, which result in extremely low densities of mosquitoes and flies, the monitoring period for these pests was set from April to November in this study, with one monitoring session conducted each month. The surveillance of rodents and cockroaches was conducted from January to December, once every odd-numbered month, with at least six times a year. All monitoring activities were scheduled to take place in the middle of each monitoring month and would be postponed in case of rainy or windy weather. All the vectors captured were taken to the laboratory, and identification was performed under the stereo microscope according to the dichotomous key or detection images (23). Among them, adult mosquitoes were additionally subjected to sex identification, and the number of female mosquitoes was recorded.

Mosquito surveillance methods. In the process of mosquito surveillance, different monitoring methods exhibit certain selectivity toward mosquito species. Since *Culex* and *Aedes* genu*s* mosquitoes are the dominant species in Zhejiang Province, the surveillance primarily focuses on these two types of mosquitoes. Adult mosquito surveillance used the CDC light trap methods, mainly for the surveillance of the mosquitoes of the *Culex* genu*s*. The places far away from the interfering light sources and sheltered from wind were chosen as the surveillance sites, and two mosquito light traps were placed 1.5 m above the ground and left overnight in each village. The power supply was connected and the CDC light trap was turned on 1 h before sunset to capture mosquitoes, and it remained on until 1 h after sunrise the next day. Mosquito larvae surveillance used the Breteau Index (BI) method, for the surveillance of the *Aedes* genu*s* mosquitoes. At least 50 households in each village were selected, distributed across the four cardinal directions (east, south, west, and north), and all the containers in or around the houses were examined for the *Aedes* larvae mosquitoes. Mosquito surveillance was conducted in the middle of each month, and if there was rain or strong winds, the specific monitoring time would be postponed.

Fly surveillance methods. Two fly traps with the bait of brown sugar, vinegar, and water (50 g + 50 g + 50 mL) were placed in each village, mainly in the green belts, dining environments, residential areas, agricultural markets, or locations where flies were likely to occur due to the abundant presence of organic matter or food sources. The traps were arranged before 9:00 a.m. in the morning on the first day and collected in 9:00 a.m. the next day.

Rodent surveillance methods. The night trapping method was used for the rodent surveillance. The medium mouse traps were distributed every 15 m^2^ indoors or every 5 meters along the wall root in rooms over 100 m^2^, and no less than 25 households were selected in each village. All the traps were placed at dusk and taken back in the next morning.

Cockroach surveillance methods. At least 50 cockroach sticky boards with 2 grams of fresh bread were placed in the farmers’ kitchen, dining room, food storage room, or locations where cockroaches were likely to occur due to the abundant presence of organic matter or food sources in each village, which were placed in the evening and collected in the next morning.

### Statistical analysis

The adult mosquito density was calculated as the number of female mosquitoes caught per trap-night. The mosquito larvae density used the BI, which was calculated as the number of positive containers for *Aedes* larvae per 100 households. The density of the flies was calculated as the number of flies per trap. The rodent density was estimated as the number of rodents caught per hundred trap-nights. The cockroach capture rate was estimated as the percentage of the positive sticky board (%). The cockroach density was estimated as the number of cockroaches caught per sticky board. All the descriptive statistics were performed using the R version 4.0.2 (The R Foundation for Statistical Computing). The normality test was performed for the mosquito, fly, rodent density, and the cockroach capture rate, in which the findings showed all the data did not satisfy the normal distribution. Thus, the Kruskal–Wallis test was used for the density comparison, with a *p*-value of ≤0.05 considered to be statistically significant.

## Results

### Results of the long-term vector density control effect

By the end of 2023, the FPFV in Zhejiang Province had reached over 900 villages, and a large number of rural residents had benefited, which was a beneficial exploration in the sustainable control of vectors in rural areas. During the study period in 2023, the temperature range in Zhejiang Province was 4.39°C to 30.00°C for the rodent and cockroach surveillance period and 11.38°C to 30.00°C for the mosquito and fly surveillance period from April to November. The relative humidity typically varied between 64.60 and 92.08%. A total of 42 villages were included in the analysis of the adult mosquito density, including 21 FPFV and 21 control villages. Overall, 698 light traps were placed in the survey villages, and 4,728 adult mosquitoes were trapped, including 2,520 female mosquitoes, with the total density of 3.61 mosquitoes per trap-night. The mosquito density of the FPFV was 2.32 mosquitoes per trap-night, which was lower than the control villages (4.87 mosquitoes per trap-night). For different mosquito species, *Culex pipiens pallens* (*Cx. p. pallens*) was the major species, which account for 91.03% of the captured mosquitoes, followed by *Aedes albopictus* (*Ae. albopictus*, 5.67%), *Culex tritaeniorhynchus* (*Cx. tritaeniorhynchus*, 1.96%), *Armigeres subalbatu*s (*A. subalbatu*s, 0.93%), and *Anopheles sinensis* (*An. sinensis*, 0.34%). For different cities, Zhoushan city had the highest mosquito density (8.81 mosquitoes per trap-night), followed by Jinhua city (5.48 mosquitoes per trap-night). Except for Zhoushan city, the mosquito densities in the FPFV were all lower than the control villages in the other 10 cities of Zhejiang Province, and a large density gap was appeared in Jinhua city and Jiaxing city (Table 2).

**Table 2:** Adult mosquito density in the villages of Zhejiang Province in 2023.

City	No. of light traps	No. of mosquitoes	No. of Female mosquitoes	Mosquito density (mosquitoes per trap-night)			*Culex pipiens pallens*		*Aedes albopictus*		*Culex tritaeniorhynchus*		*Armigeres subalbatus*		*Anopheles sinensis*		Unidentified
				Total	FPFV	Control villages	Female	Male	Female	Male	Female	Male	Female	Male	Female	Male	
Hangzhou	48	223	127	2.65	2.34	3.25	127	96	0	0	0	0	0	0	0	0	0
Huzhou	112	717	444	3.96	2.46	5.46	444	273	0	0	0	0	0	0	0	0	0
Jiaxing	64	469	217	3.39	0.59	6.19	217	252	0	0	0	0	0	0	0	0	0
Jinhua	64	777	351	5.48	0.97	10.00	271	320	80	106	0	0	0	0	0	0	0
Lishui	128	204	156	1.22	0.44	2.00	48	33	8	0	63	9	30	0	7	6	0
Ningbo	57	485	255	4.47	4.24	4.66	234	216	17	9	1	0	2	1	1	1	3
Quzhou	48	180	90	1.88	1.75	1.94	85	86	2	1	3	3	0	0	0	0	0
Shaoxing	96	852	453	4.72	4.06	5.38	442	398	11	1	0	0	0	0	0	0	0
Taizhou	32	218	117	3.66	1.44	5.88	82	88	18	11	14	0	3	2	0	0	0
Wenzhou	17	39	28	1.65	1.50	1.78	25	11	2	0	-	-	0	0	1	0	0
Zhoushan	32	564	282	8.81	9.19	8.44	277	279	2	0	0	0	3	3	0	0	0
Total	698	4,728	2,520	3.61	2.32	4.87	2,252	2052	140	128	81	12	38	6	9	7	3
Percentage (%)	-	-	-	-	-	-	47.63	43.40	2.96	2.71	1.71	0.25	0.80	0.13	0.19	0.15	0.06

A total of 41 villages were included in the analysis of the *Aedes* larvae density, including 21 FPFV and 20 control villages. A total of 11,310 water bodies in 16,283 households were monitored, and 2,320 water bodies were found *Aedes* larvae positive, with the total BI of 14.25. The BI of the FPFV was 9.70, which was obviously lower than that of the control villages (BI: 19.41). The small idle containers (36.00%) and the water deposits aquatic plants (35.77%) were the most common types of water bodies in the rural areas of Zhejiang Province. In the *Aedes* larvae positive water bodies, the small idle containers accounted for the highest proportion (54.87%). For different cities, the BI of Jinhua city was the highest (BI: 30.38). The BI of the FPFV was lower than the control villages in most of the cities in Zhejiang Province. The BI meet the criterion (BI < 5) in all the FPFV investigated in Jiaxing and Shaoxing city (Table 3).

**Table 3:** *Aedes* larvae density in the villages of Zhejiang Province in 2023.

City	No. of households	No. of water Bodies	No. of *Aedes* larvae Positive water bodies	BI			Flowerpot water		Larger water storage containers		Small idle containers		Other types of water	
				Total	FPFV	Control villages	Total number	Positive number	Total number	Positive number	Total number	Positive number	Total number	Positive number
Hangzhou	1,620	3,677	179	11.05	8.56	13.51	2,692	29	421	70	536	69	28	11
Huzhou	3,100	1,273	304	9.81	10.26	9.35	420	119	585	116	244	68	24	1
Jiaxing	800	478	125	15.63	2.00	29.25	164	25	124	21	126	48	64	31
Jinhua	1,600	896	486	30.38	6.50	54.25	167	70	79	23	600	376	50	17
Lishui	2,875	1,229	480	16.70	12.50	21.96	229	67	257	115	505	227	238	71
Ningbo	1,003	916	117	11.67	5.76	16.49	43	10	301	41	487	60	85	6
Quzhou	800	746	112	14.00	5.50	22.50	107	9	291	37	317	61	31	5
Shaoxing	880	176	84	9.55	4.55	14.55	28	20	66	23	81	41	1	0
Taizhou	404	476	76	18.81	7.35	30.50	55	5	68	12	189	49	164	10
Wenzhou	800	220	69	8.63	8.50	8.75	6	1	26	10	184	58	4	0
Zhoushan	2,401	1,223	288	12.00	14.62	6.75	134	12	202	33	803	216	84	27
Total	16,283	11,310	2,320	14.25	9.70	19.41	4,045	367	2,420	501	4,072	1,273	773	179
Percentage 1 (%)	-	-	-	-	-	-	-	15.82	-	21.59	-	54.87	-	7.72
Percentage 2 (%)	-	-	-	-	-	-	35.77	-	21.40	-	36.00	-	6.83	-

A total of 706 fly traps were distributed in 43 villages, including 21 FPFV and 22 control villages, and 1,248 flies were captured, with the total fly density of 1.77 flies per trap. The fly density of the FPFV (1.32 flies per trap) was lower than the control villages (2.17 flies per trap). The species of the *Sarcophagidae* family (31.97%) and *Musca domestica* (*M. domestica,* 31.41%) were the most common species in the rural areas of Zhejiang Province. For different cities, the fly density of Hangzhou city (4.62 flies per trap) was relatively high. The fly density of the FPFV was lower than the control villages in most of the cities in Zhejiang Province, except for Hangzhou and Zhoushan city (Table 4). For the standard of the FPFV, the fly density meet the criterion (adult fly density ≤0.5 flies/cage) in all the villages investigated in Wenzhou, Jiaxing, and Jinhua city.

**Table 4:** Fly density in the villages of Zhejiang Province in 2023.

City	No. of Fly traps	No. of Flies	Flies density (flies per trap)			No. of different species of flies									
			Total	FPFV	Control villages	Species of *Sarcophagidae* family	*Musca domestica*	*Lucilia sericata*	*Musca sorbens*	*Muscina stabulans*	*Lucilia cuprina*	*Chrysomya megacephala*	*Lucilia illustris*	*Calliphora vicina*	Others
Hangzhou	34	157	4.62	8.19	1.44	71	25	36	1	11	9	3	0	0	1
Huzhou	64	152	2.38	2.09	2.66	49	14	8	0	32	1	2	3	17	26
Jiaxing	128	138	1.08	0.13	2.03	40	82	6	0	0	0	7	0	0	3
Jinhua	64	94	1.47	0.38	2.56	54	4	23	0	10	0	0	0	0	3
Lishui	128	208	1.63	1.11	2.14	3	135	0	59	0	11	0	0	0	0
Ningbo	74	97	1.31	0.69	1.65	40	14	9	11	0	5	8	6	0	4
Quzhou	32	32	1.00	0.63	1.38	5	17	0	0	0	0	0	10	0	0
Shaoxing	66	110	1.67	1.44	1.88	22	63	22	0	0	1	0	1	0	1
Taizhou	32	50	1.56	1.44	1.69	20	3	6	10	2	1	2	0	0	6
Wenzhou	48	148	3.08	0.06	4.59	59	31	8	9	12	10	15	1	0	3
Zhoushan	36	62	1.72	2.83	0.61	36	4	0	0	6	9	1	5	0	1
Total	706	1,248	1.77	1.32	2.17	399	392	118	90	73	47	38	26	17	48
Percentage (%)	-	-	-	-	-	31.97	31.41	9.46	7.21	5.85	3.77	3.04	2.08	1.36	3.85

A total of 24,405 mousetraps were distributed, and 153 rodents were captured in 42 villages, including 22 FPFV and 20 control villages, with the rodent density of 0.63 rodents per 100 trap-nights. Rodent density of the FPFV was lower than the control villages. *Rattus norvegicus* (*R. norvegicus*) was the most common species (38.56%) in the rural area of Zhejiang Province, followed by *Rattus flavipectus* (*R. flavipectus,* 17.65%). No rodent was observed in the FPFV investigated in six cities, namely, Ningbo, Taizhou, Huzhou, Jiaxing, Shaoxing, and Hangzhou. Except for Lishui, Zhoushan, and Jinhua, the other cities all meet the rodent criterion of the FPFV (the rodent density ≤1.0 per 100 trap-nights) (Table 5).

**Table 5:** Rodent density in the villages of Zhejiang Province in 2023.

City	No. of mousetraps	No. of rodents	Rodent density (rodents per 100 trap-nights)			No. of different species of rodents						
			Total	FPFV	Control villages	*Rattus norvegicus*	*Rattus flavipectus*	*Mus musculus*	*Apodemus agrarius*	*Niviventer confucianus*	*Suncus murinus*	Others
Hangzhou	4,428	0	0.00	0.00	0.00	0	0	0	0	0	0	0
Huzhou	2,940	0	0.00	0.00	0.00	0	0	0	0	0	0	0
Jiaxing	1,040	2	0.19	0.00	0.38	2	0	0	0	0	0	0
Jinhua	2,400	67	2.79	1.25	4.33	21	17	6	2	15	6	0
Lishui	3,044	42	1.38	1.12	1.64	19	8	1	14	0	0	0
Ningbo	2,648	2	0.08	0.00	0.15	1	0	1	0	0	0	0
Quzhou	850	13	1.53	0.18	4.00	6	2	2	0	0	1	2
Shaoxing	2,398	0	0.00	0.00	0.00	0	0	0	0	0	0	0
Taizhou	691	3	0.43	0.00	0.64	2	0	0	0	0	1	0
Wenzhou	1,800	5	0.28	0.67	0.08	1	0	0	0	0	4	0
Zhoushan	2,166	19	0.88	1.11	0.64	7	0	1	0	2	7	2
Total	24,405	153	0.63	0.41	0.84	59	27	11	16	17	19	4
Percentage (%)	–	–	–	–	–	38.56	17.65	7.19	10.46	11.11	12.42	2.61

A total of 10,122 sticky boards were distributed, and 673 cockroaches were captured in 38 villages (19 FPFV and 19 control villages), with the cockroach density of 0.07 cockroaches per sticky board, and cockroach capture rate was 3.83%. The cockroach density and cockroach capture rate in the FPFV (0.03 cockroaches per sticky board, 1.77%) was lower than the control villages (0.10 cockroaches per sticky board, 5.87%), respectively. The *Blattella germanica* (*B. germanica,* 65.68%) was the most common species in the villages of Zhejiang Province, followed by *Periplaneta americana* (*P. americana,* 18.42%) and *Periplaneta fuliginosa* (*P. fuliginosa,* 15.90%). The cockroach capture rate in the FPFV of Ningbo, Lishui, Zhoushan, and Jiaxing city meet the cockroach criterion (the cockroach capture rate ≤1.0%) (Table 6).

**Table 6:** Cockroach density in the villages of Zhejiang Province in 2023.

City	No. of sticky boards	Positive sticky boards	No. of cockroaches	Cockroach density (cockroaches/sticky board)			Cockroach capture rate (%)			No. of different species of cockroaches			
				Total	FPFV	Control villages	Total	FPFV	Control villages	*Blattella germanica*	*Periplaneta americana*	*Periplaneta fuliginosa*	Others
Hangzhou	294	6	8	0.03	0.04	0.01	2.04	2.99	1.25	8	0	0	0
Huzhou	240	41	106	0.44	0.13	0.76	17.08	6.67	27.50	102	2	2	0
Jiaxing	2,400	26	34	0.01	0.00	0.03	1.08	0.17	2.00	34	0	0	0
Jinhua	1,200	176	332	0.28	0.08	0.48	14.67	4.33	25.00	144	103	85	0
Lishui	2089	52	70	0.03	0.01	0.05	2.49	0.88	4.05	67	3	0	0
Ningbo	1,625	4	4	0.00	0.00	0.00	0.25	0.29	0.21	0	4	0	0
Quzhou	358	27	39	0.11	0.08	0.17	7.54	5.04	12.50	14	11	14	0
Shaoxing	800	24	36	0.05	0.03	0.06	3.00	2.13	3.98	36	0	0	0
Taizhou	571	22	34	0.06	0.06	0.06	3.85	4.38	3.44	34	0	0	0
Wenzhou	300	6	6	0.02	0.03	0.00	2.00	3.00	0.00	3	0	3	0
Zhoushan	245	4	4	0.02	0.00	0.04	1.63	0.00	4.00	0	1	3	0
Total	10,122	388	673	0.07	0.03	0.10	3.83	1.77	5.87	442	124	107	0
Percentage (%)	–	–	–	–	–	–	–	–	–	65.68	18.42	15.90	0.00

### Density comparison

As shown in Table 7, all the densities investigated in the FPFV were significantly lower than the control villages, including the adult mosquitoes, the *Aedes* larvae, the flies, the rodents, and the cockroaches (all *p* < 0.05).

**Table 7:** Results of the density comparison between the FPFV and the control villages.

Factors	FPFV (M ± SD)	Control village (M ± SD)	*Z*	*P*
Adult mosquito density	2.43 ± 3.90	4.61 ± 5.19	−4.943	0.000
*Aedes* larvae density	7.92 ± 8.67	22.03 ± 2.56	−7.561	0.000
Fly density	1.50 ± 2.56	2.31 ± 3.16	−3.756	0.000
Rodent density	0.36 ± 0.86	1.07 ± 1.92	−2.912	0.004
Cockroach capture rate	2.51 ± 5.58	8.03 ± 18.00	−2.802	0.005

## Discussion

In recent years, with the process of rural revitalization program (24) in China, the improvement of the infrastructures and hygiene conditions provided a good opportunity for the prevention and control of the vectors in rural areas. Since the first “Mosquito-free village” began to pilot in Zhejiang Province in 2016 (15), the sustainable vector management strategies in rural areas had been practiced. By the end of 2023, more than 900 FPFV had been successful certified, benefiting thousands of rural residents. The major intention of the FPFV was that the density of the “four pests” could be controlled at an extremely low level by sustainable vector control measures, without spreading related infectious diseases and harassing people’s lives (17). However, it did not mean that no single vector could be tolerated in the villages, which was unrealistic and unsustainable. The effectiveness had been fully recognized by the local governments and the rural villagers, which had greatly improved the overall hygiene conditions of local villages, as well as increased the awareness of relevant disease prevention of local residents (25).

Based on the intention of the FPFV, formulation of the criterion was mainly aimed at improving the villages’ hygiene condition and relevant disease prevention. To ensure the authority and accuracy of the evaluation indicators, the density control indicators for mosquito, fly, rodent, and cockroach were mainly referred to the PRC National Standard such as criteria for vector density control—rodent, mosquito, fly, and cockroach (19–22). In addition, part of the vector control standards were based on the disease prevention and control needs. Research found that the BI value of 5 served as the lowest threshold, and where the BI value was > 5 with reported dengue cases or BI was > 20 even without any dengue case, control measures should be taken for the mosquito control (26), so the BI in the criterion was set to less than 5. Relevant study suggested that the rodent density controlled below 1.0 rodents per 100 trap-nights could effectively control the risk of hemorrhagic fever with renal syndrome (27), which was included in the development of the rodent density indicators in our criterion. Except for the evaluation of the “four pest” density, the overall environment of the village, the village organization management, the health education, the vector integrated control methods, the vector surveillance, etc. were all included in the evaluation criterion of the FPFV (18, 28). After all, the density control of the “four pests” was a long-term process, which required the enthusiasm of villagers to develop good hygiene habits and participating enthusiasm. All these indicators were set and assigned a certain score, and the overall score of the village investigated above 80 points could be named the FPFV. So, the vector density index was not the only index for the FPFV evaluation, which could ensure the participation of the rural residents and the sustainability of the whole project. Overall, the criterion could meet the evaluation requirements of the FPFV, but with the continuous progress, the index might need to be continuously updated.

The surveillance results found that the total vector densities of mosquito, fly, rodent, and cockroach investigated in the FPFV were all lower than the control villages, which indicated a significant vector control effect. The mosquito density control in the FPFV was highly effective, in which the adult mosquito density and mosquito larvae density were all reduced over 50% than the controls. The surveillance results found that *Cx. p. pallens* was the major species in the rural area of Zhejiang Province, which account for 91.03% of the captured mosquitoes by the CDC light trap method. Due to the limitations of the adult mosquito surveillance method we used, the CDC light trap method was not particularly sensitive to the *Ae. Albopictus.* However, mosquitoes of the *Culex* genus and *Ae. Albopictus* were the dominant mosquito species in Zhejiang Province (29). Therefore, mosquito monitoring and control efforts in Zhejiang Province had primarily been focused on these two types of mosquitoes. Due to the different ecological habits of these two types of mosquitoes, there is currently no single mosquito monitoring method that can effectively monitor the densities of both types of mosquitoes simultaneously. Thus, the BI method was added in our research for the monitoring of the *Aedes* mosquitoes. The total BI of the FPFV was 9.70, which was obviously lower than that of the control villages (BI: 19.41), but it was higher than the criterion (BI < 5). The results indicated that although the mosquito density in FPFV was significantly lower than that in the control villages, it was challenging to maintain the “mosquito-free” standard in the long term in some FPFV. Further analysis found that the small idle containers and the flowerpot water were the most common types of water bodies in the rural areas, and the small idle containers accounted for the highest positive proportion (54.87%). For the living habits of rural residents, a large number of water containers were existed around the house of the residents, which might be the reason for the high density of the *Aedes* larvae in rural area (14). Meteorological factors, particularly the temperature and relative humidity, have been reported to have substantial impacts on vector organisms. To minimize the potential confounding effects, we carefully considered these factors in our study design. Specifically, we applied the principle of 1:1 matching to select control villages within the same city or district as the experimental villages. This matching was based on several criteria, including meteorological factors, to ensure comparability between the experimental and control sites. In addition, in selecting the monitoring time for this study, we took into account of the local climatic conditions and seasonal fluctuations in vector population densities. Mosquito surveillance was conducted in the middle of each month. However, if there is rain or strong wind, the monitoring time will be postponed to avoid biased results due to adverse weather conditions. These measures were taken to ensure that the density data collected were as representative as possible.

For the fly surveillance, the species of *Sarcophagidae* family and *M. domestica* were the major species in the rural areas, which was basically consistent with the study of Wu et al. (30). In our study, the species of *Sarcophagidae* family was identified only at the family level. This family is highly diverse and complex, with many species sharing similar ecological habits and disease transmission potential. Given the challenges in differentiating individual species within this family, and considering that their monitoring and control methods are largely similar, we adhered to the standardized protocol implemented by the Chinese CDC since 2016 (31). This protocol focuses on broader ecological and public health implications rather than individual species differentiation and thus does not require identification of *Sarcophagidae* beyond the family level. The fly density control in the FPFV was also effective, and the fly density of the FPFV was lower than the control villages in most of cities in Zhejiang Province. Our results showed that the pilot experience of the FPFV in rural areas had provided a good opportunity for the breeding areas removal and density control of the mosquito and fly. It indicated the environmental governance combined with certain physical or biological control measures could control the density of the mosquito and fly at a relatively lower level.

Due to the presence of the external environment such as farmland in the rural areas, the rodent density control remained a difficult issue. The comprehensive rodent control measures including environmental management measure, physical control measure, and chemical control measure were all used in the FPFV. Practices proved that these measures were scientifically effective, and the rodent density of the FPFV was lower than the control villages. No rodents were observed in the FPFV in six cities, namely, Ningbo, Taizhou, Huzhou, Jiaxing, Shaoxing, and Hangzhou. Except for Lishui, Zhoushan, and Jinhua city, the other cities all meet the rodent criterion of the FPFV. *R. norvegicus* was the major species, followed by *R. flavipectus*, which was slightly different from previous studies in Zhejiang Province (29). An ongoing surveillance study in Zhejiang Province found that *R. norvegicus* was the major species, followed by *M. musculus*, *S. murinus*, and then *R. flavipectus* (13). This study only included the rural residential area as the habitat, and the slight difference might be due to the difference of the habitats investigated. The cockroach control in rural areas was also a major concerning problem, which could also focus on environmental control, combining physical and biological control, and taking appropriate chemical control measures. Practices had proved that the effect of this measure was remarkable, and the cockroach density and cockroach capture rate in the FPFV were lower than the control villages. Study found that the infestation trend of *B. germanica* was increasing (32), and our research found that *B. germanica* was one of the most important cockroach species to be controlled in rural areas, which accounted for 65.68% of the cockroaches captured. Although using symbiotic microorganisms for the biological control of the cockroach was found (33), it was difficult to large-scale promotion and use. The *B. germanica* had strong reproductive ability, adaptability, and resistance to certain insecticides, so the cockroach control in rural areas remained a challenge, and only four cities investigated all meet the criterion of the FPFV.

All the pest surveillance results showed that the densities of mosquitoes, flies, rodents, and cockroaches in the FPFV were all lower than those in the control villages, while the pest densities in some villages cannot consistently met the FPFV criterion in long term. There might be several reasons for this. One reason was that during the evaluation of FPFV, the scoring mechanism requires only 80 points to pass, which allows some flexibility in the control level of pest densities. The most likely reason was that, despite a significant increase in community engagement after being designated as a FPFV, the importance of pest control in these villages gradually decreased over time. Therefore, to sustain low pest densities in FPFV, continuous and active community participation was necessary. While chemical insecticides can rapidly reduce the density of the “four pests” to a low level, this method is not sustainable. Chemical control is highly specialized, difficult for villagers to master, and also expensive. Only by enhancing the participation of the villagers, combining daily organizational management and vector density monitoring work, could the density of the “four pests” be continuously controlled at a relatively low level. A questionnaire survey was conducted in 2021 in Zhejiang Province, which found that 98.92% of the villagers investigated support the pilot program of the FPFV, 99.46% of the villagers were satisfied with the pilot effect, and 98.39% of the villagers expressed their willingness to cooperate with the pilot of the FPFV (25). It indicated that the FPFV pilot methods and related standards were approbatory by the local government and villagers. The FPFV was an innovative and effective work of the patriotic health movement in rural areas.

Our study had several strengths. This is one of the few studies that systematically described a new exploration on sustainable vector management strategies in rural areas of Zhejiang Province, namely, FPFV, and summarized the pilot experience, the relevant criterion, and the long-term actual vector density control effect. In addition, this study is representative in Zhejiang Province with the monitoring sites we selected which covered all the 11 prefecture-level cities. Meanwhile, several limitations of this study should be acknowledged. First, the CDC light trap method was used to investigate the adult mosquito density, while the criterion of FPFV used the landing index method to evaluate the adult mosquito density. Thus, it was unclear whether the adult mosquito density of FPFV met the criterion. Second, this study lacked the investigation and analysis of villagers’ cognition and satisfaction for the FPFV. Although the villagers’ awareness and satisfaction had been assessed before FPFV was named, whether it has long-term effects remains to be seen and needs to be further explored in future studies. Third, the study’s evaluation was based on a limited sample, raising concerns about the generalizability of the findings to broader rural areas of Zhejiang Province and other regions with different ecological and socioeconomic contexts. In addition, the primary outcome measure was vector density, but other analyses were lacking. Specifically, the study did not compare results with baseline data or analyze the impact of meteorological factors, which would have provided a more comprehensive understanding of changes over time. Moreover, pathogen detection was not performed on the collected vector specimens, especially blood-feeding mosquitoes, and the indicator of vector-borne diseases incidence was not assessed. This limited the evaluation of their potential role in disease transmission and the impact on human health. Future research should adopt uniform methods and expand the sample size to improve the reliability and applicability of the findings. Meanwhile, incorporating baseline data comparisons, meteorological factor analyses, and assessments of vector-borne disease incidence, as well as employing molecular biological techniques for pathogen detection, would contribute to a more thorough evaluation.

## Conclusion

The findings suggested that the community-based sustainable control method of vectors, namely, the FPFV, is relatively practical and reliable and also is suitable for the rural villages. The pilot projects focused on the problems of vector organisms in rural areas and carried out sustainable vector control activities through scientific means adhering to the principles of green, economic, and sustainable. Through a series of measures such as village organization management, health education, villagers mobilization, breeding ground clearance, vector control, and vector density evaluation, the vector density in the village can be controlled at an extremely low level for a long time, greatly reducing the harassment of vector species on the population and the spread of related diseases.

## Data Availability

The raw data supporting the conclusions of this article will be made available by the authors, without undue reservation.
